# Supplementary material for: Pregnant women’s knowledge of obstetrical danger signs: A cross-sectional survey in Kigali, Rwanda
Source: PLOS Glob Public Health. 2022 Nov 14;2(11):e0001084. doi: 10.1371/journal.pgph.0001084 (PMC10021381; doi:10.1371/journal.pgph.0001084)
Supplement: Attachment — Submitted filename: Response to Reviewers.pdf [file pgph.0001084.s003.pdf]

Emmanuel Uwiringiyimana
University of Rwanda/ CMHS
Kigali, KK503 Road

Email: emma6as@gmail.com

Tel: +250788977117

August 26th, 2022

Dear Chief editor of PLOS- Global Public health

I am replying in response to the reviewer's comments on my original research article entitled **"Pregnant women's knowledge of obstetrical danger signs: A cross-sectional survey in Kigali, Rwanda,"** previously submitted and regarding the response to feedback given to me.

General response

After seeing the reviewers' comments, we decided to do major revisions to our manuscript. We revised the background, rerun the analysis using the latest software (Stata/BE 17.0) and this time conduct multiple logistic regression to address any potential confounders in assessing factors associated with maternal knowledge of ODS, and revised the discussion according to the revised findings.

Comments

2. Does this manuscript meet PLOS Global Public Health's publication criteria? Is the manuscript technically sound, and do the data support the conclusions? The manuscript must describe methodologically and ethically rigorous research with conclusions that are appropriately drawn based on the data presented.
Reviewer #1: No

- **Dear reviewer, thanks for the insightful comment. We have revised the manuscript according to the journal publication criteria, and you will find that the content is relevant and accurate along with the conclusion in line with the objectives of our study.**

3. Has the statistical analysis been performed appropriately and rigorously?
Reviewer #1: No

- **Thank you, reviewer #1, for the comment. In the revised analysis, we used multiple logistic regression to assess the factors associated with the knowledge of ODS. This analysis adjusted for all sociodemographic and pregnancy history factors. Our choice of factors to include in the model was informed by multiple systematic reviews on health literacy. We conducted our analysis in the latest Stata/BE 17.0 software.**

4. Have the authors made all data underlying the findings in their manuscript fully available (please refer to the Data Availability Statement at the start of the manuscript PDF file)? The PLOS Data policy requires authors to make all data underlying the findings described in their manuscript fully available without restriction, with rare exception. The data should be provided as part of the manuscript or its supporting information, or deposited to a public repository. For example, in addition to summary statistics, the data points behind means, medians and variance measures should be available. If there are restrictions on publicly sharing data—e.g., participant privacy or use of data from a third party—those must be specified.
Reviewer #1: No

- **Dear reviewer, we appreciate your comment and the time you took to write it here.**
- **We agree that it is important to make all data underlying the findings described in their manuscript fully available without restriction. Therefore, we have provided the dataset in the previous revision.**

5. Is the manuscript presented in an intelligible fashion and written in standard English?PLOS Global Public Health does not copyedit accepted manuscripts, so the language in submitted articles must be clear, correct, and unambiguous. Any typographical or grammatical errors should be corrected at revision, so please note any specific errors here.
Reviewer #1: No

- **During our redaction of our revisions, we have paid attention to the grammar. We have used the premium version of Grammarly (a software that detects errors in English grammar) to help detect errors in our latest draft.**

6. Review Comments to the Author Please use the space provided to explain your answers to the questions above. You may also include additional comments for the author, including concerns about dual publication, research ethics, or publication ethics. (Please upload your review as an attachment if it exceeds 20,000 characters)

Reviewer #1: It is a bit unclear to me why this study is relevant for an international readership given that similar studies were conducted already in three other countries. The study is also limited to the capital city, in the sample size, and using descriptive methods. The underlying causal link between knowing a danger sign and maternal death needs to be strengthened. It is possible that a woman cannot name a danger sign but still acts when she is bleeding or in severe pain. Thus I found that important to emphasise that if they are unsure about these signs they might delay action. Also please refer here to the fact that seeking healthcare is costly that is why they need to be more sure when to act. Stating that maternal death is high worldwide is not true. It is quite low in high-income settings which further emphasises that this would be mostly preventable. The discussion needs to be better structured. Also, you draw conclusions that I believe are not supported by your findings such as "Encouraging the involvement of husbands in all perinatal visits at health centers and hospitals has the potential to improve outcomes, as well as encouraging legal marriage". You did not really investigate this issue. =This being said I agree with you but it's not your conclusion). The sample size is rather small and does not allow multivariate analysis. Sample size should be mentioned in the abstract. You state that the inclusion criterion was 'aged 18 years and older' but you 11 participants under 18 in Table 1. It is unclear to me how you asked about danger signs. Was it an open-ended question or a closed question? Maybe women cannot name blurred vision as a problem by themselves but when you asked them whether it is

a problem, they will name it as a problem. Please specify how you exactly asked about these and possibly make the questionnaire available. There are some typos

- **Thank you for the comment.**

This study is fit for a global audience because this study is the first of its nature to be conducted in Rwanda. Implementers (local and international), or researchers could refer to these findings while planning interventions aiming to reduce maternal mortality. It is essential that interventions towards maternal health do not leave out health literacy during pregnancy.

Our introduction clearly describes that the lack of knowledge of ODS along with the high costs of care could lead to hesitation in seeking care hence poor health outcomes, including maternal mortality.

We revised the discrepancy in the age description. To clarify, we included participants aged 16 and above.

While the study is limited in external validity to the urban population of Kigali, this is the initial step to assessing maternal health literacy on pregnancy matters.
With 382 samples, we have 80% power to detect odds ratio of 1.5 and above with 50/50 proportion distribution.

The questions were mostly multiple choice. While asking questions about knowledge of ODS, the participants were to select the correct danger signs from a pool of correct and wrong responses.

After conducting the analysis and adjusting for all sociodemographic and pregnancy history factors, age, attendance to ANC, marital status, and religion became significantly associated with the knowledge of ODS. We restructured our discussion and conclusion based on these new findings.

Let us know if there are additional questions about this manuscript and send them to emma6as@gmail.com.

Thank you for your consideration of this manuscript.

Sincerely,

EmmaU.

Emmanuel Uwiringiyimana
