# Supplementary material for: Pregnant women’s knowledge of obstetrical danger signs: A cross-sectional survey in Kigali, Rwanda
Source: PLOS Glob Public Health. 2022 Nov 14;2(11):e0001084. doi: 10.1371/journal.pgph.0001084 (PMC10021381; doi:10.1371/journal.pgph.0001084)
Supplement: Attachment — Submitted filename: Rebuttal letter (response to reviewers).doc [file pgph.0001084.s002.doc]

Emmanuel Uwiringiyimana
University of Rwanda/ CMHS
Kigali, KK503 Road

Email: [emma6as@gmail.com](mailto:emma6as@gmail.com)

Tel: +250788977117

2^nd^ January, 2022

Dear, Chief editor of PLOS- Global Public health

I am replying to the response to the review comment of my original research article entitled **“Pregnant women's knowledge of obstetrical danger signs: A cross-sectional survey in Kigali, Rwanda**” previously submitted to PLOS- Global Public health.

**Comments**

1. Please provide additional details regarding participant consent. In the ethics statement in the Methods and online submission information, please ensure that you have specified whether consent was informed.

We have added the statement about consent on line 144-146

“Participation in the study was voluntary. Pregnant mothers received the explanation of the study's intent, what their participation will entail. Pregnant women who agreed to participate signed the consent form prior to initiating the interview. The collected data was kept confidential on a Password Protected laptop only accessible to the research team prior to the deidentification.”

2. Please provide separate figure files in .tif or .eps format only, and remove any figures embedded in your manuscript file.

We removed figures from the manuscript. We prepared images in a using .tiff format and checked them in PACE. We uploaded them in figures as compressed folder called archive

3. Please update the completed 'Competing Interests' statement, including any COIs declared by your co-authors. If you have no competing interests to declare, please state "The authors have declared that no competing interests exist". Otherwise please declare all competing interests beginning with the statement "I have read the journal's policy and the authors of this manuscript have the following competing interests:"

We added the statement “The authors have declared that no competing interests exist". On the line 325

4. In the online submission form, you indicated that "The collected data was kept confidential, on a Password Protected laptop Only accessible to the research team prior to the deidentification" All PLOS journals now require all data underlying the findings described in their manuscript to be freely available to other researchers; either 1. In a public repository, 2. Within the manuscript itself, or 3. Uploaded as supplementary information.

provided the dataset in .csv format and uploaded it as a supplementary file

5. If you did not receive any funding for this study, please simply state: "The authors received no specific funding for this work”.

We specified that we received no funding for this work. On the line 326

6. Other “concerns about whether this study provides new information with global implications, warranting publication in the Plos Global Public Health.

While the studies exploring maternal knowledge of obstetrical danger signs exist, this study is the first of a kind conducted in Rwanda. While the funding and capacity (being the student project) did not permit recruitment of country representative sample nor a fair representation of rural and urban participants – this study serves as baseline and provides vital information for improving maternal pregnancy outcomes and future research in this area.

Let us know if there are additional questions about this manuscript and send them to [emma6as@gmail.com](mailto:emma6as@gmail.com) .

Thank you for your consideration of this manuscript.

Sincerely,

Emmanuel Uwiringiyimana
